# Decoding viral protein sequences by large language models

**DOI:** 10.1093/bib/bbag377

**Published:** 2026-07-17

**Authors:** Tianyi Fei, Siqi Li, Ziyue Yang, Kaitao Zhou, Yixue Li, Tao Zeng

**Affiliations:** GMU-GIBH Joint School of Life Sciences, Guangdong Provincial Key Laboratory of Protein Modification and Disease, The Guangdong-Hong Kong-Macao Joint Laboratory for Cell Fate Regulation and Diseases, Guangzhou Medical University, Guangzhou 511436, Guangdong Province, China; Guangzhou National Laboratory, No. 9 XingDaoHuanBei Road, Guangzhou International Bio Island, Guangzhou 510005, Guangdong Province, China; School of Life Sciences and Technology, ShanghaiTech University, No. 393 Middle Huaxia Road, Shanghai 201210, China; GMU-GIBH Joint School of Life Sciences, Guangdong Provincial Key Laboratory of Protein Modification and Disease, The Guangdong-Hong Kong-Macao Joint Laboratory for Cell Fate Regulation and Diseases, Guangzhou Medical University, Guangzhou 511436, Guangdong Province, China; Guangzhou National Laboratory, No. 9 XingDaoHuanBei Road, Guangzhou International Bio Island, Guangzhou 510005, Guangdong Province, China; Guangzhou National Laboratory, No. 9 XingDaoHuanBei Road, Guangzhou International Bio Island, Guangzhou 510005, Guangdong Province, China; School of Life Sciences and Technology, ShanghaiTech University, No. 393 Middle Huaxia Road, Shanghai 201210, China; Guangzhou National Laboratory, No. 9 XingDaoHuanBei Road, Guangzhou International Bio Island, Guangzhou 510005, Guangdong Province, China; School of Automation Science and Engineering, South China University of Technology, 381 Wushan Road, Tianhe District, Guangzhou 510641, Guangdong Province, China; GMU-GIBH Joint School of Life Sciences, Guangdong Provincial Key Laboratory of Protein Modification and Disease, The Guangdong-Hong Kong-Macao Joint Laboratory for Cell Fate Regulation and Diseases, Guangzhou Medical University, Guangzhou 511436, Guangdong Province, China; Guangzhou National Laboratory, No. 9 XingDaoHuanBei Road, Guangzhou International Bio Island, Guangzhou 510005, Guangdong Province, China; GMU-GIBH Joint School of Life Sciences, Guangdong Provincial Key Laboratory of Protein Modification and Disease, The Guangdong-Hong Kong-Macao Joint Laboratory for Cell Fate Regulation and Diseases, Guangzhou Medical University, Guangzhou 511436, Guangdong Province, China; Guangzhou National Laboratory, No. 9 XingDaoHuanBei Road, Guangzhou International Bio Island, Guangzhou 510005, Guangdong Province, China

**Keywords:** large language models, protein language models

## Abstract

Large language models (LLMs) for biological sequences are transforming computational biology, enabling a nuanced understanding of protein and nucleotide sequence data. Recent models, including ESM2, ESM3, AlphaGenome, Evo-1, and Evo-2, adapt natural language processing principles to the biological domain by learning high-dimensional hidden representations that capture evolutionary constraints, structural patterns, and functional motifs. This mini-review summarizes recent developments in devising and applying such models, emphasizing viral protein analysis. We highlight studies that have leveraged sequence-based LLMs in the protein domain (i.e. protein language models, or PLMs) for important application tasks such as viral protein annotation, variant effect prediction, and immune escape characterization. Additionally, we present a benchmark evaluation of these state-of-the-art protein language models to evaluate their core ability to capture evolutionary relationships between viral protein sequences. By discussing the opportunities and challenges of PLMs, the review outlines a road map for the potential application of LLMs in empowering virology research and pathogen surveillance.

## Introduction

Viral proteome evolution is a complex, multidimensional process involving transmissibility, immune escape, and mutational epistasis. Traditional statistical and machine learning methods have difficulty capturing high-dimensional sequence features’ nonlinear relationships and long-range dependencies, and their predictive accuracy is often limited [1]. In this regard, next-generation artificial intelligence (AI) technologies hold great promise for deciphering viral evolutionary patterns and predicting mutation risks [2–4], and large language models (LLMs) based on the transformer architecture have provided a revolutionary new paradigm in recent years. Specifically, they can decode viral sequences’ deep biological logic and integrate multiple evolutionary information sources, including sequence conservation, structural constraints, and functional phenotypes. When combined with fine-tuning of LLMs on viral sequence data from specific scenarios, AI models’ predictive accuracy far surpasses that of traditional approaches.

**Figure 1:**
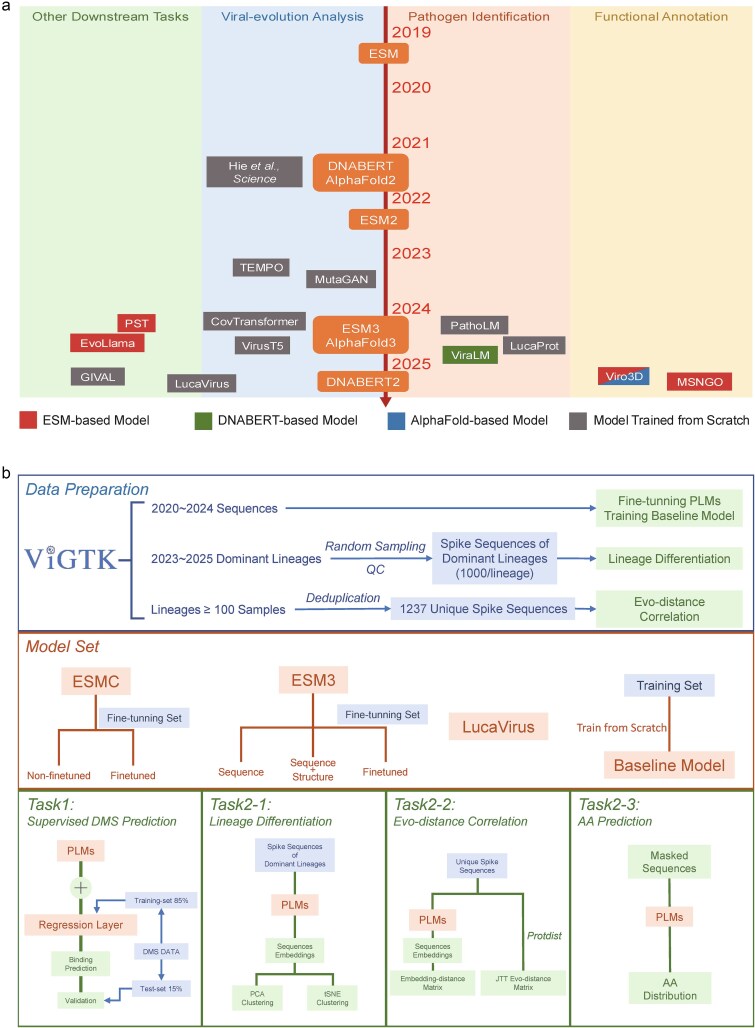
Overview of sequence-based LLMs for viral research and the benchmark design for comparing different PLMs. (a) Timeline and landscape of representative large biological sequence models applied to virology-related tasks, whose implement details and model features can be found in Tables 1 and 2. The panel here summarizes key models and methods from 2019 to 2025 across four major application areas: Viral-evolution analysis, pathogen identification, functional annotation, and other downstream tasks. (b) Schematic of the ViGTK data preparation and model benchmarking framework. Top: Data preparation pipeline. Middle: Model settings for benchmark. Bottom: Downstream four benchmark tasks. The details of benchmarking can be found in method setup.

Large biological sequence models are derived from LLMs based on biological sequence data (Fig. 1a and Table 1). Since the introduction of the transformer architecture in 2017 [5], its core self-attention mechanism has been adapted to the biological domain [6, 7]. This transfer has enabled numerous tools for processing nucleic acid and protein sequences. Currently, foundation models based on biological sequence are undergoing rapid development and iteration, evolving from earlier nucleic acid sequence models (e.g. GenSLMs [8], megaDNA [9], and EVO [10]) and protein sequence models (e.g. ProteinBERT [11] and ESM2 [12]) to new models that can handle multimodal data (e.g. LucaOne [13] and ESM3 [14]). These LLMs in the biological domain have already achieved significant breakthroughs in diverse downstream tasks (Table 2), including protein structure prediction and antibody design [15–18].

**Table 1:** Summary of LLMs in recent biological studies.

**Type of sequences**	**Model**	**Year**	**Architecture**	**Training dataset**
Nucleotide models	GenSLMs	2023	Transformer (decoder-only) + stable diffusion	Pretrained on BV-BRC prokaryotic genes and fine-tuned on BV-BRC SARS-CoV-2 genomes
	megaDNA	2024	Three layers of decoder-only transformer (local–middle–global) with multi-head attention	~99.7 K high-quality bacteriophage genomes from NCBI GeneBank, MGV, and GPD
	Evo	2024	Hybrid architecture of decoder-only transformer++ and Hyena	>80 000 bacterial and archaeal genomes and millions of predicted phage and plasmid sequences (OpenGenome)
	ViraLM	2024	Transformer (encoder-only)	Virus dataset and host dataset
	DNABERT	2021	Transformer (encoder-only)	Sampled human reference genome
	DNABERT-2	2023	Transformer (encoder-only)	Human genome + 153 other species genomes
	PathoLM	2024	Transformer (encoder-only)	~49.6 k PATRIC bacterial genomes and ~25 k viral genomes
	Nucleotide transformer	2023	Transformer (encoder-only)	3202 human genomes and 850 genomes from other species
Protein models	ESM	2019	Transformer (encoder-only)	UniRef50
	ESM2	2022	Transformer (encoder-only)	UniRef/metagenomic sequences
	ESMFold	2022	Transformer (encoder-only)	UniRef/metagenomic sequences
	Viro3D	2025	A pipeline that integrates AlphaFold2, ESMFold, Foldseek, and other structure analysis tools	Proteomes from >4400 human and animal viruses
	TEMPO	2023	Transformer (encoder-only)	~7.4 million spike protein sequences from GISAID with phylogenetic trees from RCov19, and HA sequences from H1N1, H3N2, H5N1
	MutaGAN	2023	Seq2seq GAN: LSTM-based generator paired with discriminator	6840 HA sequences of H3N2 (from NCBI Influenza Virus Resource)
	VirusT5	2025	Transformer (encoder–decoder)	Segmented genomes (<512 bps) of 100 000 SARS-CoV-2 strains
	CovTransformer	2024	Transformer-based linear regressor	Metadata for SARS-CoV-2 sequences from GISAID
	GIVAL	2025	Semi-supervised pipeline that combined a encoder-only transformer and a ResNet classifier	~1.86 million viral protein sequences from 42 viral families
	LucaProt	2024	Dual-channel transformer (encoder-only)	≈6 k RdRP and ≈229 k non-RdRP
	LucaVirus	2025	RoFormer (encoder-only)	~15.7 million viral sequences

**Table 2:** Characteristics of LLMs in recent biological studies.

**Model**	**Training strategies**	**Evolution relevant**	**Pathogen relevant**	**Function relevant**	**Other abilities**
GenSLMs	Transformer was trained using autoregressive modeling and next a diffusion model was trained to capture long-range genome context	√	×	×	×
megaDNA	Autoregressive training	√	√	√	×
Evo	Autoregressive training	√	√	√	Genome-level generation; design of mobile genetic elements and CRISPR-Cas systems
ViraLM	Fine-tuned on DNABERT-2	×	√	×	×
DNABERT	MLM on K-mer tokenized Sequences, de novo training	×	√	√	×
DNABERT-2	MLM on BPE tokenized Sequences, de novo training	×	√	√	×
PathoLM	MLM fine-tuned on pretrained model (Nucleotide Transformer v2 50M)	√	√	×	×
Nucleotide transformer	MLM for pretraining and LoRA for fine-tuning, de novo training	×	√	√	Potential in capturing effect of synonymous and intergenic variants
ESM	MLM, de novo training	√	×	√	Secondary structure prediction
ESM2	MLM, de novo training	√	√	√	Structure prediction
ESMFold	MLM, trained on ESM2	√	×	√	Structure prediction
Viro3D	×	√	√	√	Structure prediction
TEMPO	TEMPO was trained as a supervised binary classifier based on phylogenetic tree and embedding with ProtVec	√	×	×	Predict future mutations
MutaGAN	Adversarially trained on parent–child pairs that generator generate child sequences and discriminator validate these sequences	√	×	×	Predict future mutations
VirusT5	MLM, de novo training	√	√	×	Predict future mutations
CovTransformer	Supervised trained on lineage frequency regression	×	×	×	Lineage frequency forecasting and pandemic monitoring
GIVAL	The integrated transformer was trained with MLM approach, and the ResNet was trained with flexible labels	×	√	×	Host adaptation prediction of divers viruses
LucaProt	Supervised training, de novo training	×	√	√	×
LucaVirus	MLM, based on LucaOne	√	√	√	Prediction of Ag-Ab binding affinity

While LLMs have developed rapidly in the field, several challenges persist. First is the inevitable homogeneity of training data and the limited use of high-quality labeled or supervised data. Especially in the field of viral proteins, significant shortcomings remain in terms of data resources. The core difficulties lie in the dearth of high-quality annotated data and systematic evolutionary trajectory maps. Moreover, viral datasets with complete spatiotemporal evolutionary chains are extremely rare. Of note, the mutations observed for some highly conserved proteins so far are also extremely limited, which makes it is difficult to learn their evolutionary patterns. Second, most LLMs, specifically protein language models (PLMs), focus on a single protein or region and consider the sequence as a single modality, which typically ignore important information such as the protein structure. This restricted scope prevents models from learning co-evolutionary relationships between different viral proteins, thereby confining their predictive capability to individual proteins. This limitation can lead to a narrower focus and insufficient information intake and can cause inherent limitations when handling complex downstream tasks such as predicting viral virulence or the properties of virus–host immune interactions. Until recently, these PLMs have been evaluated in several existing benchmarks. For instance, ProteinGym [19] has set the gold standard for predicting mutational fitness, and PFMBench [20] has extensively assessed general capabilities in structural and functional annotation [21]. However, a critical gap remains within the specific context of viral evolution. Current benchmarks rarely evaluate whether a model’s latent space aligns with phylogenetic relationships or assess performance in lineage differentiation.

Given the opportunities and challenges presented by sequence-based LLMs, this mini-review highlights studies that leverage these models for key viral protein analysis tasks. Additionally, we provide a benchmark evaluation of several representative PLMs to assess their practical performance in fine-tuning of models. To that end, we assessed the essential responses of PLMs to viral protein sequence evolution and developed a road map for the LLMs’ wider application potential in virology research and pathogen surveillance.

## L‌LMs reshaping pathogen identification and functional annotation

The viral functional annotation and prediction field is transitioning from classical homology-based workflows to multimodal foundation-model paradigms. However, a key bottleneck in this transition is the generally low quality of viral genome annotation in metagenomic datasets, which curtails both viral diversity and functional potential. Because metagenomic sequencing often produces numerous short reads and viral contigs are of low abundance in complex communities, traditional similarity-based tools produce substantial noise when assigning viral identity to fragmentary sequences.

Based on the LLMs’ principle, ViraLM [22], built on the genomic language model DNABERT-2 [23], adopts a pretraining fine-tuning regimen that improves robustness in recognizing short and highly divergent viral sequences. In RNA virus research, Hou *et al*. [24] integrated evolutionary sequence features with structural conservation to develop the multimodal model LucaProt, thereby transcending the limitations of global sequence alignment. By focusing on key functional domains of the viral RNA-dependent RNA polymerase (RdRP), LucaProt enables systematic discovery of previously unrecognized RNA virus lineages from diverse environmental and host-associated metagenomes. For public health applications, PathoLM [25] leverages the nucleotide transformer [26] to perform cross-species pathogenicity prediction directly from DNA, and its few-shot learning capability offers an early-warning tool for assessing the risks of emerging viruses.

At the protein-function level, synergy between PLMs and structural biology has substantially improved functional annotations’ interpretability. For example, Flamholz *et al*. [27] demonstrated that PLM embeddings capture deep functional similarity across taxonomic boundaries, expanding annotation coverage for prokaryotic virus proteins (e.g. bacteriophages), which can even generate credible biological hypotheses for novel functional modules and in combination with structure modeling. Based on AlphaFold2-ColabFold and ESMFold, Ulad Litvin *et al*. [28] constructed the Viro3D database to collect and visualize the predicted structures of viral proteins, thereby expanding the coverage of viral protein structures by 30-fold. This data resource supports cross-species evolutionary analyses and provides a structure-based context for drug target discovery.

Furthermore, Wang *et al*. [29] fused ESM2 [12] sequence representations with predicted-structure contact-graph features within a heterogeneous network propagation framework, thereby improving multi-species, multi-label Gene Ontology (GO) prediction. Although this study did not directly evaluate viral proteins, the “sequence/structure multimodality + network propagation” paradigm offers a viable strategy for functional transfer in virology and a blueprint for integrating structural repositories (e.g. Viro3D) with ecological and interactome networks.

## L‌LMs empowering viral-evolution analysis and many downstream tasks

Recent advances in LLM architectures and scale, along with sustained community investment, have driven rapid progress in diverse virus-prediction tasks. The COVID-19 pandemic further intensified this trend by greatly expanding genome surveillance and the volume of publicly available sequence data, which enable more ambitious site-, sequence-, and population-level modeling efforts.

At the site level, distinguishing stochastic variation from adaptive substitution requires integrating the local sequence context with temporal and phylogenetic history. One early representative study framed escape prediction with a language-model perspective [30] by implementing a Bidirectional Long Short-Term Memory (BiLSTM) network to score grammaticality (viability) and semantic change (functional or antigenic shift) across influenza hemagglutinin (HA), HIV Env, and SARS-CoV-2 Spike. Building on the same intuition that temporal context matters, later methods incorporated explicit phylogenetic paths. For example, TEMPO samples evolutionary paths from a phylogenetic tree and feeds them into a transformer encoder to predict per-site substitution probabilities [31]. These methods upgrade static frequency counts to time-sensitive risk estimates that are useful for experimental prioritization, however they typically do not specify exact substitution types or fully capture high-order epistatic interactions.

As attention shifted to combinations of mutations at the sequence level, generative models became prominent. In one sense, sequence-to-sequence Generative Adversarial Networks (Seq2Seq-GANs) and conditional transformer architectures can simulate higher-order epistatic interactions among multiple sites, and propose plausible, but not yet observed sequences. For example, MutaGAN is a Seq2Seq-GAN that simulates influenza HA evolution and reproduces certain historical mutation patterns [32]. However, its outputs require downstream biological scoring to exclude non-viable candidates. In another sense, mutation prediction can be treated as a translation task in a related paradigm, where a parental genome is the source text and its likely descendants are the target. For example, conditional transformer models trained on ancestor–descendant sequence pairs within variants of concern (VOC) (e.g. VirusT5) can learn within-VOC evolutionary trajectories and generate candidate descendants [33]. The above generative methods are powerful for candidate proposal but must be combined with structure- and function-aware filters to reduce false positives.

Furthermore, at the population level, lineage or variant-frequency forecasting addresses operational public-health questions such as “which lineage is likely to dominate in the coming weeks?” Time-series transformers that take in recent daily or weekly frequencies, sequencing depth, and spatiotemporal metadata can predict near-term frequency trajectories and provide lead time for surveillance. CovTransformer exemplifies this approach by modeling lineage frequency series with attention-based architectures to predict future prevalence [34]. Such methods are directly useful for monitoring and resource allocation. However, they are sensitive to sampling bias and rely on well-defined lineage annotations.

In addition to the above three core axes, parallel downstream application tasks address host-adaptation prediction and protein-property inference. Host-adaptation frameworks typically couple pretrained protein encoders with supervised classifiers. For instance, the GIVAL pipeline uses a Bidirectional Encoder Representation from Transformers (BERT)-style protein encoder to derive embeddings, applies clustering and label refinement, and trains a ResNet classifier to score adaptation across influenza, coronavirus, and orthopoxvirus proteins [35]. Moreover, protein-level property prediction (stability, solubility, localization, and interaction propensity) has benefited from multimodal and fine-tuned pretrained models. EvoLlama, a hybrid system that embeds protein sequences with large protein models (ESM2) and fuses those embeddings with language models for contextual metadata (Llama), can improve downstream physicochemical and interaction predictions [36]. Moreover, architectures such as the protein set transformer treat a viral genome as a set of protein embeddings and aggregate them with set or graph transformer modules to produce genome-level representations for comparative genomics and functional annotation [37].

Indeed, a major methodological shift underlies these expansions. The research and application field has shifted from task-specific, from-scratch training toward a pretrain-then-fine-tune paradigm. Pretraining on large protein or genomic corpora yields general representations that reduce labeled-data requirements and improve cross-task and cross-species transfer. Then, task-specific heads and supervised fine-tuning adapt these hidden representations for site prediction, sequence generation, frequency forecasting, and protein property regression. In practice, operational surveillance systems are expected to adopt modular pipelines that chain candidate generators, biology-aware scorers (structure/stability/epitope overlap), and population-level forecasters, which can aid in biologically filtering and epidemiologically monitoring of relevant variants.

Collectively, research on viral evolution and variant surveillance has developed from single-site detection to integrated systems that propose candidates, assess biological plausibility, and forecast population impact. Realizing robust, operational systems requires carefully fusing pretrained representations with explicit temporal, phylogenetic, and structural constraints, alongside rigorous validation against molecular assays and epidemiological outcomes.

## Opportunities and challenges of sequence-based LLMs: Insights from benchmarking

To achieve a deeper quantitative understanding of the opportunities and challenges of sequence-based LLMs, we undertook a benchmarking analysis and discussion based on our designed supervised and unsupervised tasks for three state-of-the-art (SOTA) PLMs (Fig. 1b): ESM3, ESMC, and LucaVirus. For ESM3 and ESMC, we incorporated Low-Rank Adaptation (LoRA) parameters to fine-tune the models for these tasks. For LucaVirus, we directly used the embeddings it provides for downstream tasks, based on its original pretrain version specifically on viral sequences. Additionally, a small transformer model trained from scratch (i.e. a five-layer transformer with RoPE embeddings, 8 M parameters) was employed as a baseline method. Specifically, we retrieved 19 431 818 global Spike sequences from ViGTK covering the period 1 January 2020 through 12 July 2025, which were mainly from the USA (34.9%) and the UK (21.6%). Through performance comparison, we identified the opportunities and the challenges of applying sequence-based LLMs, especially to viral protein analysis.

**Figure 2:**
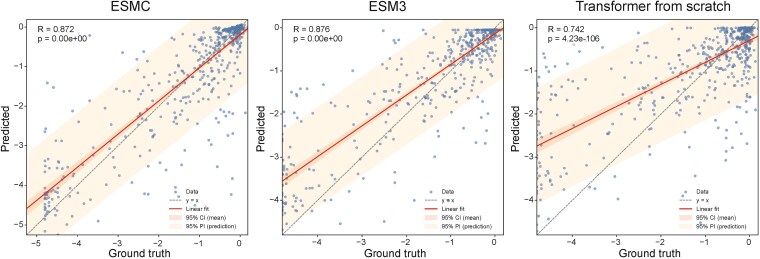
Performance of predicted versus experimentally measured binding affinity (DMS scores) for single–amino acid mutations in the SARS-CoV-2 Spike protein based on DMS fine-tuning task**.** The compared models include ESMC (left), ESM3 (middle), and a small transformer trained from scratch (right). ESMC and ESM3 achieve remarkable correlation between predicted and experimentally measured DMS scores, which outperform baseline (i.e. transformer from scratch). This suggests pretraining can enhance generalization in supervised tasks (e.g. DMS fine-tuning task here), which would be attributed to the inductive transfer and the learning of evolutionary constraints.

### Benchmark setup

(1) Task 1 (deep mutational scanning [DMS] fine-tuning): In this supervised task, we used a DMS dataset measuring the binding affinity between SARS-CoV-2 Spike and ACE2, comprising 4003 variant sequences. The data were split into 15% held-out test sequences and 85% remaining sequences, with the latter further divided into 85% training and 15% validation. For both ESM3 and ESMC, we fine-tuned using a parameter-efficient strategy by adding LoRA adapters (rank = 4) to all layers. Of note, we controlled redundancy through subsampling during training instead of applying explicit global deduplication to the Spike sequence collection. Each training run uses a fixed-size subset (10 k sequences per run) drawn from the full dataset, where the time-and-geography—–uniform sampling can better reflect the temporal and regional diversity of surveillance data.
(2) Task 2 (Masked language modeling fine-tuning, i.e. MLM fine-turning): In this unsupervised task of studying evolutionary modeling at scale, we sampled Spike sequences uniformly across time and geography from a ~20 million sequence ViGTK collection. Specifically, we first selected countries with more than 500 000 available sequences, then sampled 100 sequences per week to construct a training set of 196 526 sequences. During training, 15% of residues in each input sequence were randomly masked following the standard MLM objective, and models were fine-tuned using LoRA adapters (rank = 4) added to every layer. Training was run for 10 000 iterations using a cross-entropy loss to predict the masked amino acids. For downstream analyses, we used the mean-pooled representation of the model’s final layer (averaged over sequence length) as the sequence embedding.
(3) Downstream-analysis 1 related to task 2 (Lineage differentiation by embedding-based clustering): To evaluate whether PLM embeddings capture epidemiologically meaningful lineage structure, we made selection of the lineage with the highest prevalence for each month from 2023 onward, where the lineages whose Spike proteins contained no novel mutations were excluded. For each retained lineage, we collected sequences from its month of peak prevalence, applied quality control (removing sequences with ≥5% deletions or ambiguous amino acids in the Spike protein), and then randomly sampled 1000 Spike sequences per lineage. For each sequence, we constructed a kNN graph in embedding space (using *k* = 15), then applied Leiden community detection with resolution = 0.2 to obtain unsupervised clusters. We quantified agreement between predicted clusters and the ground-truth lineage labels using five standard external clustering metrics: Adjusted Rand Index (ARI) measures pairwise agreement between cluster assignments and labels with chance correction; Normalized Mutual Information (NMI) measures mutual dependence between clusters and labels normalized to [0,1]; Adjusted Mutual Information (AMI) is the chance-corrected version of mutual information; Fowlkes–Mallows Index (FMI) evaluates the geometric mean of pairwise precision and recall for “same-cluster” decisions; and Homogeneity (HOM) measures cluster purity (each cluster contains sequences primarily from a single lineage), noting that homogeneity alone does not penalize splitting a lineage into multiple clusters. These metrics quantify the agreement between the ground-truth lineage labels and the clusters formed by the embeddings, with values closer to 1.0 indicating better separation.
(4) Downstream-analysis 2 related to task 2 (Embedding–alignment distance correlation): To assess whether embedding geometry reflects evolutionary relatedness, we screened ViGTK and retained 1237 SARS-CoV-2 lineages that contained at least 100 sequences. For each lineage, we derived a single representative Spike sequence using a consensus approach. We then extracted embeddings for these representative sequences using each model and computed pairwise cosine distances in embedding space. In parallel, we computed pairwise evolutionary distances between the representative sequences from sequence alignments using a Jones-Taylor-Thornton (JTT)-corrected distance model. Finally, we quantified the agreement between embedding-derived distances and alignment-based evolutionary distances using Pearson correlation (linear association) and Spearman correlation (rank-order association), respectively.
(5) Downstream-analysis 3 related to task 2 (Whole-Spike scanning): To quantify residue-level mutation preferences, we performed position-wise masking by masking one site at a time across the full Spike sequence and using either the pretrained or fine-tuned model to predict the amino acid probability distribution at that masked position. The predicted distribution was then compared with the empirical amino acid distribution estimated from the full ~20 million-sequence dataset at the same position, and we used KL divergence to quantify the discrepancy between the model-predicted and population-derived distributions. A lower Kullback-Leibler (KL) divergence indicates higher similarity between two distributions.

### Enhanced generalization: Pretraining in supervised task

Our supervised regression task, e.g. Task 1 (DMS fine-tuning), predicts the binding affinity between the SARS-CoV-2 Spike protein and ACE2 using DMS data [38] as a gold-standard. In a DMS dataset, the effect of each site mutation in the Spike protein was experimentally measured. We fine-tuned the models for this regression task (Fig. 2) and observed that pretrained PLMs (i.e. ESM3 model with Pearson correlation of regression values as 0.876) significantly outperformed the baseline (i.e. small scratch-trained model with Pearson correlation of regression values as 0.742). This leads to our understanding that the pretraining on massive biological corpora provides a robust initialization that is superior to directly learning from scratch.

From a computational perspective, the baseline model would suffer from the “cold-start” problem. Without pretraining, it must simultaneously learn the fundamental grammar of protein sequences and the specific regression task from a limited DMS dataset. As known, in the DMS regression setting, the effect of a mutation on Spike–ACE2 binding is largely governed by two biological factors: (i) the position being mutated (i.e. whether it lies within or near binding-relevant regions); and (ii) the specific amino acid substitution, especially how different the substituted residue is from the original one in terms of physicochemical properties. Thus, the model trained from scratch would perform poorly on a subset of mutations that are expected to have large impacts, frequently predicting only minor effects.

Actually, the above superior performance of pretrained PLMs (ESM3, ESMC) over the scratch-trained baseline in the DMS fine-tuning task (Fig. 2) can be attributed to the inductive transfer and the learning of evolutionary constraints. The pretrained PLMs leverage transfer learning to provide informative priors about amino acid similarity and protein domain relationships. As demonstrated by Meier *et al*. [39], the pretraining objective (Masked Language Modeling) forces PLMs to capture the underlying evolutionary landscape, where probability serves as a proxy for fitness. These priors likely enable the model to more accurately localize ACE2-binding relevant regions and to better evaluate whether a given substitution constitutes a substantial property change. Consequently, the pretrained embeddings implicitly encode structural constraints and functional viability, thereby improving the prediction of large-effect mutations even when the labeled DMS training set in task is limited. Obviously, the scratch-trained model lacks such “evolutionary intuition,” resulting in its inability to distinguish between deleterious and neutral mutations effectively.

### Latent semantics: Lineage and evolutionary distance

**Figure 3:**
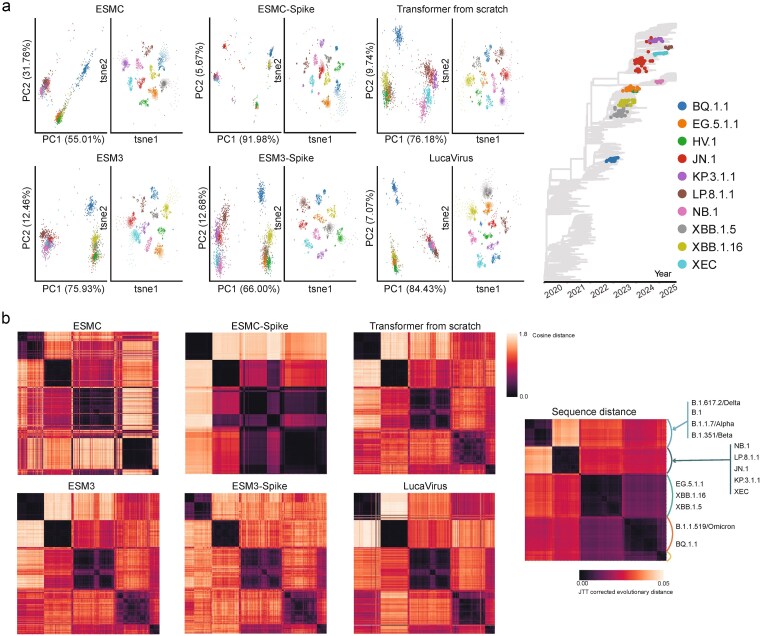
Protein language models capture SARS-CoV-2 lineage structure and evolutionary distance based on MLM fine-tuning task. (a) Lineage differentiation by embedding-based clustering. Two-dimensional projections of Spike protein embeddings for 10 recent SARS-CoV-2 lineages (1000 sequences per lineage) generated by different PLMs: Pretrained ESMC and ESM3, their Spike-only fine-tuned variants (ESMC-Spike, ESM3-Spike), LucaVirus model, and baseline model (i.e. a small transformer trained from scratch). Points are colored by Pango lineage, and axes show the first two principal components (and corresponding explained variance) or t-SNE dimensions. These results have shown that the learned hidden representations (i.e. embeddings) are interpretable. For example, PCA projections of PLM embeddings partition sequences in a manner consistent with known phylogeny, with closely related lineages forming compact clusters and more distant lineages occupying distinct regions of embedding space. (b) Embedding–alignment distance correlation. For 1237 SARS-CoV-2 lineages from ViGTK (≥100 sequences per lineage), we computed pairwise cosine distances between lineage-mean Spike embeddings and compared them with JTT-corrected evolutionary distances derived from multiple sequence alignments. Such qualitative and quantitative results demonstrate that PLM embeddings can encode biologically meaningful evolutionary information and explain why their geometry characteristics can interpret lineage relationships.

Our unsupervised task, e.g. Task 2 (MLM fine-turning), naturally capture evolutionary information in latent embeddings; however, their application to viral surveillance is complicated by data homogeneity. We assessed such challenge in two different downstram analysis: Lineage differentiation using embeddings (Fig. 3a and Table 3) and Embedding alignment based on distance correlation (Fig. 3b and Table 4).

**Table 3:** Performance comparison of PLM in the analysis task of lineage differentiation using embeddings according to five clustering metrics.

**Model**	**ARI**	**NMI**	**AMI**	**FMI**	**HOM**
ESMC	**0.9001**	**0.9143**	**0.9139**	**0.9117**	0.9652
ESMC-Spike	0.8713	0.8951	0.8944	0.8871	0.9666
ESM3	0.8750	0.8964	0.8957	0.8897	0.9640
ESM3-Spike	0.8846	0.9107	0.9102	0.8990	0.9805
LucaVirus	0.8773	0.8985	0.8974	0.8927	**0.9813**
Transformer from scratch	0.8556	0.8983	0.8978	0.8738	0.9694

**Table 4:** Performance comparison of PLM model in the analysis task of embedding–alignment distance correlation based on Pearson correlation (*r*) and Spearman correlation (*ρ*), respectively.

**Model**	**Pearson *r***	**Spearman *ρ***
ESMC	0.508056	0.416551
ESMC-Spike	**0.866363**	**0.878455**
ESM3	0.796673	0.740814
ESM3-Spike	0.815410	0.786098
LucaVirus	0.749940	0.680977
Transformer from scratch	0.796673	0.740814

The PLMs achieved good performance, even without fine-tuning (Downstream-analysis 1 in Fig. 3a and Table 3), indicating that pretraining already captures aspects of evolutionary information such as the biological distance between sequences (Downstream-analysis 2 in Fig. 3b and Table 4). The results further shows that these learned hidden representations (i.e. embeddings) are interpretable (Fig. 3a). principal components analysis (PCA) projections of PLM embeddings partition sequences in a manner consistent with known phylogeny, where closely related lineages form compact clusters and more distant lineages occupy distinct regions of embedding space. For example, BQ.1.1 (a BA.5 descendant) is positioned apart from the other nine sampled lineages; among those nine, EG.5.1.1, HV.1, XBB.1.5, and XBB.1.16 (XBB-derived) group together, whereas JN.1, KP.3.1.1, NB.1, XEC, and LP.8.1.1 (BA.2.86 descendants first observed in 2023) form a separate cluster. As known, XBB.1.5, XBB.1.16, EG.5.1.1, and HV.1 began to progressively replace BA.5 and its descendants as the globally dominant lineages starting in February 2023. Meanwhile, JN.1, KP.3.1.1, XEC, LP.8.1.1, and NB.1 (BA.2.86 descendants) subsequently rose in prevalence from January 2024 and have since become the new dominant global lineages. Further, cosine distances in embedding space correlate strongly with JTT-corrected evolutionary distances, with the highest correspondence observed within proximate lineage groups (Fig. 3b). Therefore, the learned embeddings can encode within-lineage property similarity and between-lineage property divergence, corresponding to observed lineage replacement dynamics in the real world. Together, these qualitative and quantitative results demonstrate that PLM embeddings actually capture biologically meaningful evolutionary signals and that their geometry information can be used to interpret lineage relationships.

### Overfitting trap: High data redundancy leads to temporal memorization

**Figure 4:**
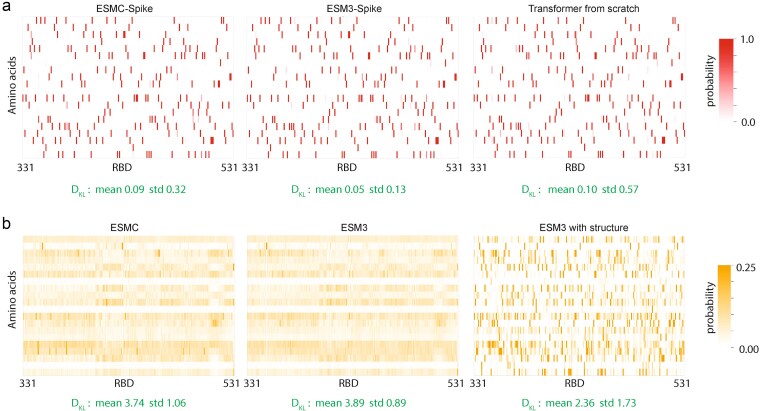
Existing challenges and opportunities observed in Whole–Spike scanning based on MLM fine-tuning task. (a) Predicted amino acid distributions after MLM fine-tuning on Spike sequences for ESMC-Spike, ESM3-Spike, and baseline method (i.e. a small transformer trained from scratch). Their performance illustrates the issue of "overfitting" where PLMs memorize the training set distribution, which might impair the ability to predict novel mutations, as the model assigned zero probability to amino acids not seen during training. Heatmaps show predicted marginal probabilities for ESMC, ESM3, and ESM3 with Spike structure provided as an additional input. (b) Position-wise amino acid distributions predicted by non–fine-tuned PLMs for the SARS-CoV-2 Spike protein in a zero-shot manner. As shown, ESM3 with the Spike protein structure as an additional input could substantially improve the predicted amino acid distributions compared to sequence-only inference. Considering the pretrained ESM3 model without any downstream parameter updates, the results here are actually independent of fine-tuning strategies, which can attribute the performance difference solely to the input modality (Sequence vs. Sequence + Structure). Heatmaps show predicted marginal probabilities for ESMC, ESM3 (without Spike structure input), and ESM3 with structure.

To further investigate the changes before and after unsupervised learning of Task 2 (MLM fine-turning), we masked each position sequentially and predicted the corresponding amino acid distribution in Downstream-analysis 3. A clear pattern of temporal overfitting (which includes many factors related to conventional types of data, model, and learning overfitting) emerged: After MLM fine-tuning, all models perfectly output the amino acid distribution appeared in the training set (Fig. 4a). Our training set only used Spike sequences before 2025 and we observed that the novel mutations of 2025 year (e.g. Q493E, T22N, F59S, R190S, V445R, which have a frequency > 10% in 2025 year) all have a prediction probability <0.1% for all models. Such type of data overfitting impaired the ability to predict novel mutations, as the model assigned zero probability to amino acids not seen during training.

This result also indicates a critical challenge related to type of learning overfitting: viral surveillance datasets, such as those for SARS-CoV-2 or influenza, often have low information density with many duplicated or near-duplicated sequences. This high sequence similarity can cause conventional MLM fine-tuning to converge rapidly and then overfit, limiting the ability of PLMs to fully exploit the evolutionary signal present in large-scale sequence collections.

The integration of structural information might help alleviate the above data overfitting from model viewpoint. This is because protein structures are evolutionary more conserved than sequences and evolve at a much slower rate [14, 40]. Thus, multimodal priors should ground predictions in stable physical constraints rather than transient historical sequence statistics, thereby preventing the model from indiscriminately memorizing past data.

### Breaking the modality barrier: From single sequences to biological systems

Indeed, current PLMs face the architectural limitations regarding modality. We focused on non-fine-tuned PLMs, e.g. ESM3, as a multimodal model, which can integrate sequence and structural information. As shown in Fig. 4b, ESM3 with the Spike protein structure as an additional input could substantially improve the predicted amino acid distributions compared to sequence-only inference. This result underscores the necessity of multimodal approaches. Of note, most current models focus on single proteins (e.g. Spike protein) due to the prohibitively expensive quadratic computational cost of transformer architecture processing entire viral proteomes (often >10 000 amino acids), which prevents models from learning co-evolutionary relationships between different genomic regions (epistasis). To advance beyond single-protein prediction, it is necessary to integrate structural data or extend context windows to capture the full "viral grammar," including virus–host interactions and genome-wide co-evolution.

## Discussion and conclusion

Compared to traditional sequence alignment frameworks, PLMs have capability to significantly enhance sensitivity in identifying remote homology relationships. Especially, in scenarios involving low sequence identity, such as the detection of remote homologs and “sequence dark matter,” the contextual representations learned by PLMs can effectively capture implicit structural and functional semantics. For instance, Dense Homolog Retriever (DHR) [41] integrates PLM representations with deep dense retrieval to achieve efficient remote homology detection without explicit sequence alignment. Furthermore, PLMSearch [42] balances engineering scalability with sensitivity. It achieves retrieval speeds comparable to MMseqs2 [43], enabling second-level retrieval for million-scale queries while tripling the sensitivity for remote homology detection. Besides, pLM-BLAST [44] demonstrates that similarity metrics derived from model learning hold the potential to replace classic substitution matrices, such as BLOSUM62, as the core component of next-generation sequence comparison. In this regard, next-generation AI approaches offer promise for deciphering viral evolutionary patterns and predicting mutation risks [2–4]. Actually, transformer-based LLMs have introduced a new paradigm in addition to conventional methods based on sequence alignment in recent years. Thus, we present a focused benchmark of PLMs that complements existing general evaluations. By specifically testing SOTA models on their ability to reconstruct evolutionary distances and distinguish SARS-CoV-2 lineages, we provide unique insights into their applicability for real-time virology research.

Most benchmarks were conducted on a single NVIDIA RTX 4090 (24 GB). All fine-tuning experiments used LoRA with rank = 4, and we now note that the additional trainable parameters and memory overhead scale approximately linearly with LoRA rank. ESM3 is the most memory-demanding model in our benchmark and exceeds 50 GB in our training setup; thus, ESM3 fine-tuning was performed on an NVIDIA H800 GPU. For Spike-only evolutionary modeling (MLM fine-tuning on our Spike sequence dataset), training converged in ~2 h with about 1500 iterations (around 12 000 sequences where the cross-entropy loss for masked locations fell to under 0.01). Such result and observation related to saturation indicate that learning variance decreases quickly and that simply adding more redundant sequences is not the bottleneck; instead, careful training control should be more important for preserving generalization. For the supervised regression task as DMS fine-tuning, training required approximately ~5 h under our settings. Taken all, these experiments are protein-centric (the evolution modeling of a single protein), which is not very difficult for model convergence. Thus, increasing PLM scale did not yield large gains for this single-protein mutation learning regime (e.g. ESMC-300 M performed similarly to ESMC-600M), suggesting that moderate-scale PLMs might be sufficient for many practical viral surveillance settings.

Supervised tasks (e.g. DMS fine-tuning task here) with limited training data can benefit disproportionately from PLMs/LLMs. When the labeled dataset is small (e.g. DMS measurements available for only a subset of mutations), the data alone may be insufficient to capture general biochemical constraints. In this regime, pretrained models provide strong prior knowledge—such as amino acid physicochemical properties and context-dependent substitution preferences—which can substantially improve sample efficiency and generalization compared with training from scratch. We therefore frame future work around improving sample efficiency and robustness in low-label regimes, including better parameter-efficient fine-tuning and evaluation on held-out mutation types or temporally separated variants.

In contrast, unsupervised or zero-shot tasks (e.g. MLM fine-tuning task here) can often perform lineage differentiation and provide useful proxies for fitness-related signals directly from embeddings or likelihood-based scores without additional training, achieving strong performance in many scenarios. However, unsupervised fine-tuning on a single protein (especially on highly redundant surveillance datasets) should be performed cautiously, as aggressive adaptation can lead to overfitting and reduced robustness to novel mutations or out-of-distribution variants. We therefore motivate future work incorporating structural signals and other contextual metadata (e.g. experimentally characterized sites, immune pressure context, temporal dynamics) to better connect model predictions to biological causality and improve robustness to novel variants.

To further overcoming overfitting caused by homogeneity and redundancy in viral surveillance data, several practical directions can be considered [45]: (a) haplotype-level deduplication, which compresses duplicated sequences while preserving counts/weights to retain prevalence information; (b) lineage/profile-based training with soft labels, where sequences are clustered into lineages and the model is trained against per-position amino acid frequency distributions rather than one-hot targets, increasing effective supervision per update and reducing distribution “collapse” to near-zero probability for unseen but plausible residues [46]; and (c) increasing diversity by incorporating more distantly related sequences and/or multi-protein training, which can reduce over-specialization to a single highly homogeneous protein dataset [4]. Moreover, additional independent information sources (e.g. temporal dynamics and lineage metadata) and more informative labels should be integrated to maintain the PLMs’ advantage under realistic surveillance conditions.

The transition from task-specific training to a “pretrain-then-fine-tune” paradigm indeed can offer a new and robust way for analyzing viral protein evolution. However, realizing the full potential of PLMs in such tasks still requires moving beyond simple MLM fine-tuning and sequence-only inputs. As our evaluation indicates, the future roadmap depends on addressing the data-efficiency and modality gaps. PLMs must evolve from single-protein predictors to genome-scale, multimodal frameworks [47] that integrate structural constraints [48], phylogenetic history [49], and temporal dynamics [50]. We anticipate that the next generation of viral foundation models based on PLMs will not only learn the “language” of protein sequences but also the “grammar” of viral replication and host interaction, ultimately enabling real-time, high-precision forecasting of wide, and diverse pandemic risks.

**Key Points**

- Prior knowledge acquired during pretraining of protein language models such as amino acid similarity and protein domain relationships helps overcome small dataset limitations and enhances generalization of large language model (LLMs).
- The severe homogeneity of virus training data and the limited use of high-quality labeled data calls for additional sources of information (e.g. temporal dynamics, lineage metadata) and novel LLM training or fine-tuning strategies.
- Rich, multi-modality inputs (e.g. structural data and other contextual information) could help fully exploit protein language model capabilities and support LLMs in empowering virology research and pathogen surveillance.

## Data Availability

No new datasets have been produced and utilized in this paper. Benchmark dataset can be accessed from ViGTK Virus Genome Toolkits for SARS-CoV-2 (https://www.biosino.org/ViGTK/). Code availability: https://github.com/tyfei0216/virus_review_bib.git
